# Insulin Hypersensitivity Induced by Hepatic PTEN Gene Ablation Protects from Murine Endotoxemia

**DOI:** 10.1371/journal.pone.0067013

**Published:** 2013-06-25

**Authors:** Philipp M. Guenzl, Roman Raim, Julia Kral, Julia Brunner, Emine Sahin, Gernot Schabbauer

**Affiliations:** Institute for Physiology, Center for Physiology and Pharmacology, Medical University of Vienna, Vienna, Austria; DRFZ, Germany

## Abstract

Sepsis still remains a major cause for morbidity and mortality in patients. The molecular mechanisms underlying the disease are still enigmatic. A great number of therapeutic approaches have failed and treatment strategies are limited to date. Among those few admitted for clinical intervention, intensive insulin treatment has proven to be effective in the reduction of disease related complications in critically ill patients. Insulin effectively reduces glucose levels and thereby contributes to protection. On the other hand insulin is a potent signaling pathway activator. One of those is the PI3K signaling axis. Activation of PI3K is known to limit pro-inflammatory gene expression. Here we can show that in a mouse model of insulin hypersensitivity induced by the deletion of the PI3K antagonist PTEN, specifically in hepatic tissue, significant protection is conferred in murine models of lethal endotoxemia and sepsis. Acute inflammatory responses are diminished, glucose metabolism normalized and vascular activation is reduced. Furthermore we investigated the hepatic gene expression profile of relevant anti-inflammatory genes in PTEN deficient mice and found marked upregulation of PPARγ and HO-1. We conclude from our data that insulin hypersensitivity via sustained activation of the PI3K signaling pathway exerts protective effects in acute inflammatory processes.

## Introduction

Acute inflammatory processes associated with septic complications result in high morbidity and mortality. Sepsis is still a major burden for our health care system. Survival rates range from 30–50%. Sepsis and its severe form septic shock occur predominantly in elderly people, with a peak in the sixth decade of life. Available effective therapeutic strategies are scarce. It is therefore imperative to search for efficient therapeutic interventions in order to prevent the exacerbated host immune response in septic patients diagnosed with systemic inflammatory response syndrome (SIRS) [1]–[3].

Sepsis leads to a dysregulated insulin/glucose metabolism with exceedingly high glucose levels contributing to tissue damage and organ failure. Insulin resistance is one of the hallmarks of sepsis leading to supra-physiologic plasma glucose levels and hyperglycemia. Enhanced glucose release, reduced glucose consumption and increased free fatty acids indicate a catabolic state in sepsis, which is further exacerbated by the release of cytokines such as TNFα (cachectin) [4]. Hyperglycemia is associated with increased coagulation [5], increased inflammatory responses and apoptosis [6]. On the other hand insulin exerts potent anti-inflammatory actions in critically ill patients and promotes vascular protection [7]–[9]. Insulin is a potent activator of the PI3K/Akt signaling pathway in order to translocate glucose transporters in insulin-sensitive tissue to the cell surface. Simultaneously insulin-activated PI3K/AKT regulates glucose metabolism, glycogen/lipid and protein synthesis and gene activation required for growth and differentiation [10].

Previously we could determine that pharmacologic inhibition of the PI3K signaling pathway enhanced pro-coagulant and pro-inflammatory gene expression *in vivo*. This was confirmed by functional data through rapid activation of the coagulation cascade and increased mortality due to the dysregulated inflammatory response [11]. Furthermore we provided evidence for a protective role of insulin in a murine endotoxemic shock model in a PI3K dependent manner [12]. In human patients it could be shown that intensive insulin therapy adjusting plasma glucose levels from hyper-glycemic to normo-glycemic levels in critical care patients significantly reduced morbidity and mortality [13]. However it is still unclear whether insulin exerts protective effects beyond simply maintaining glucose homeostasis.

Here we observed in a PTEN-dependent insulin-hypersensitive mouse model that sustained activation of PI3K in hepatic tissue reduces the inflammatory burden and normalizes the glucose metabolism by the activation of anti-inflammatory mechanisms. Specific PTEN deficiency in hepatic tissue, which is induced by Alb cre expression in PTEN^fl/fl^ mice, led to significant insulin hypersensitivity. In models of acute inflammation and sepsis, which is induced by injection of two different concentrations of *E.coli* LPS or cecal ligation and puncture (CLP), we found increased survival of male as well as female hepatic PTEN deficient mice as compared to littermate controls. Analysis of inflammatory parameters revealed reduced cytokine expression and diminished endothelial cell activation in an early phase of the disease. On a molecular level we found significant upregulation of anti-inflammatory genes, which might contribute to the protective phenotype we observed in the hepatic PTEN deficient mice.

## Methods

### Mice

All mice were bred in a SPF facility with constant temperature and a 12 h/12 h day/night cycle. Floxed PTEN mice were kindly provided by Tak W. Mak (University of Toronto, Toronto, Canada) [14]. Albumin cre mice were kindly provided by Harald Esterbauer (Medical University of Vienna, Vienna, Austria). Specificity of the cre line was confirmed by Alb cre specific PCR. Breedings with floxed PTEN cre positive and cre negative mice were arranged so that littermates could be used for all experiments. Mice deficient for p85α were kindly provided by Shigeo Koyasu (Tokyo, Japan). All mice are on C57Bl/6 background and backcrossed for at least 10 generations. Genotyping was performed at the time of weaning 3–4 weeks after birth. All animal studies were approved and comply with institutional guidelines (BMWF-66.009/0103-C/GT/2007 and BMWF-66.009/0241-II/3b/2011).

### Genotyping

Mice were earmarked and DNA was isolated from ear tissue in a proteinase K lysis buffer and subjected to direct PCR using GoTaq Polymerase (Promega). The following primers were used for genotyping: flPTEN_fwd CTCCTCTACTCCATTCTTCCC, flPTEN_rev ACTCCCACCAATGAACAAAC, Albcre_fwd ATGAAATGCGAGGTAAGTATGG, Albcre_rev CGCCGCATAACCAGTGAAAC.

### Endotoxemia

After overnight fasting PTEN^fl/fl^ Alb cre positive mice and littermate controls were subjected to a mouse model of endotoxemia that consists of intraperitoneal injection of a low dose (10 mg/kg) and a high dose (15 mg/kg) of LPS (*Escherichia coli* serotype O111:B4; Sigma). Mice were monitored for survival every 4 h. Criteria for the termination of the experiment according to the approved protocol for animal studies are: refused intake of water and food, apathy, seizures, disturbed equilibrium sense and >20% loss of weight. Survival and clinical signs of disease (0 = healthy; 4 = moribund) of mice were monitored for up to 96 h.

To achieve PI3K inhibition Wortmannin (0.06 mg/kg) was injected retro-orbitally 2 h before LPS in 200 uL of Ringers solution containing 10% (v/v) dimethyl sulfoxide (DMSO) (Sigma). Blood was collected from the retro-orbital sinus in sodium citrate (3%) 5′, 10′, 30′, 120’ and 6 h post LPS challenge for subsequent analysis.

### Polymicrobial Peritonitis/Cecal Ligation and Puncture (CLP)

PTEN^fl/fl^ Alb cre positive mice and littermate control mice have been anesthetized and a 1.5–2 cm midline incision was made through the linea alba. The cecum was located and tightly ligated at its base without causing bowel obstruction. The cecum was punctured once (21 gauge). A small amount of stool was extruded to ensure wound patency. The cecum was then placed in its original position within the abdomen. Immediately after surgery mice will receive a subcutaneous injection of 0.5 ml warm saline, placed under a heating lamp and constantly monitored until recovery. Survival and clinical signs of disease (0 = healthy; 4 = moribund) of mice were monitored for up to 96 h.

### Immunoblotting

Tissues were harvested and homogenized in Laemmli buffer, diluted with PBS and heated at 95°C for 5 min. Samples were separated on a 10% denaturing polyacrylamide gel and run at 150 V for 90 min. Separated proteins were electroblotted onto an Immobilon-P PVDF transfer membrane (Millipore) at 300 mA for 1 h. After blocking with 5% milk, the membrane was incubated over night with the primary antibody of choice (1∶1000 in milk). After washing in PBST, the membrane was incubated with the secondary antibody conjugated to HRP (1∶5000 in milk) for 2 h. Finally, membranes were incubated with SuperSignal West Femto and exposed in the FluorChem HD2 chemiluminescence imager (Alpha Innotech Corp.) for 1–20 seconds. Bands were analyzed according to their molecular weight. The following antibodies have been used: rabbit anti-PTEN (Cell Signaling Technology), rabbit anti-β-Actin (Sigma).

### ELISA

Tissues were harvested and homogenized in RIPA lysis buffer. Blood samples were harvested from the *Vena Cava* into sodium citrate (3%) and centrifuged to obtain plasma. ELISA DuoSets (R&D Systems) for IL-6, TNF-α, E-Selectin and KC/IL-8 were used according to the protocol. Briefly, the plates were coated with the antibody of choice, blocked and the diluted samples together with the standards were loaded and incubated overnight. After washing, the plates were loaded with Streptavidin-HRP and incubated for 30 min. After washing, developing solution from the Substrate Reagent Pack (R&D Systems) was added and the plates incubated for 5–10 min. The reactions were stopped by the addition of 0.5 M H_2_SO_4_ and the plates read at 450 nm in an ELISA plate reader.

### Quantitative Real-Time RT-PCR

Tissues were harvested and homogenized in Trizol Reagent (Invitrogen). RNA was isolated according to the protocol, dissolved in Nuclease-free water and stored at −80°C. cDNA was prepared from 1 µg RNA using 10× RT buffer, MgCl_2_, Oligo d(T)16 primer, RNase Inhibitor and MuLV Reverse Transriptase (all Applied Biosystems). qPCR was performed using Fast SYB Green Master Mix (Applied Biosystems) on the StepOne Real-Time PCR System (Applied Biosystems). Samples were assayed in duplicates and only analysed when the melting curves showed the desired amplicon. Transcription levels of target genes were normalized to GAPDH levels, and depicted as fold induction of unstimulated macrophages.The following primers were used for qPCR: Pten_fwd ACACCGCCAAATTTAACTGC, Pten_rev TACACCAGTCCGTCCCTTTC, cre_fwd TCGCGATTATCTTCTATATCTTCA, cre_rev GCTCGACCAGTTTAGTTACCC, Pparg1_fwd GCGGCTGAGAAATCACGTT, Pparg1_rev TCAGTGGTTCACCGCTTCTT, Pparg2_fwd CACCAGTGTGAATTACAGCAAATC, Pparg2_rev AGCTGATTCCGAAGTTGGTG, Pgc1a_fwd CCGATCACCATATTCCAGGT, Pgc1a_rev GTGTGCGGTGTCTGTAGTGG, F4/80_fwd CTTTGGCTATGGGCTTCCAGTC, F4/80_rev GCAAGGAGGACAGAGTTTATCGTG, HO-1_fwd AGGTCAAGCACAGGGTGACA, HO-1_rev CATCACCTGCAGCTCCTCAA, Gapdh_fwd GGTCGTATTGGGCGCCTGGTCACC, Gapdh_rev CACACCCATGACGAACATGGGGGC.

### Metabolic Parameters

Metabolic parameters were measured at the Department of Laboratory Medicine, Medical University of Vienna.

### Glucose Tolerance Test

After overnight fasting, blood was drawn from PTEN^fl/fl^ Alb cre positive mice and littermate controls to obtain baseline levels. Then these mice were injected with dextrose (2 mg/kg) i.p. Blood samples were taken 30′, 60′ and 120’ post injection. Blood glucose levels were measured using a standard glucometer.

### Statistical Analysis

Data were analyzed using an unpaired two-tailed Student’s t-test. Survival data were compared by a log-rank test and plotted in a Kaplan-Meier curve. Statistical analysis was performed using GraphPad Prism 4 software. Two way ANOVA analysis was used to analyze two groups over time. Values are expressed as mean ± standard deviation. Criteria for significance was p<0.05 throughout this study.

## Results

### Conditional PTEN Deficiency in Liver using Alb cre Mice Leads to Hepatomegaly and Efficient PTEN Protein Reduction; LPS does not Reduce Liver PTEN Levels

**Figure 1:**
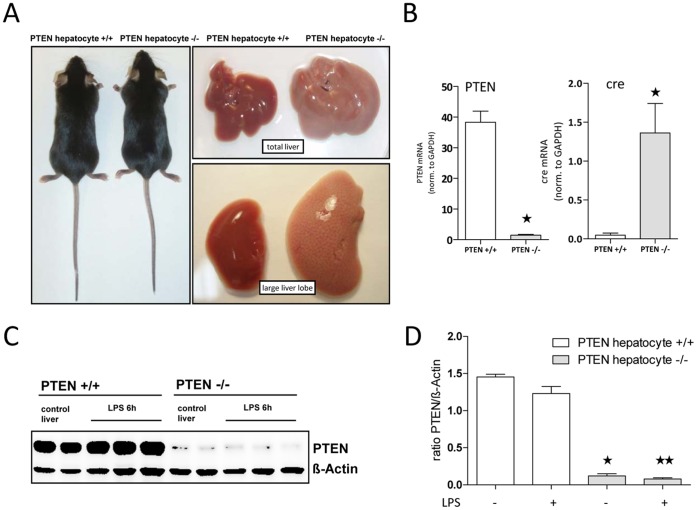
PTEN^fl/fl^ Alb cre positive mice are PTEN deficient in hepatic tissue. (A) Total liver and the large liver lobe were removed from untreated animals. A representative picture is shown. (B) RNA was extracted from naïve livers of mice. qRT-PCR was performed to measure PTEN and cre transcripts. (C) Western blotting on lysates derived from LPS treated mice detecting PTEN is shown. Actin was used as loading control. A representative picture for 3 independent experiments (each n = 3) is shown. (D) Quantification of immunoblots for PTEN expression normalized to Actin expression. *indicates p<0.05.

To study potential effects of Insulin and Insulin hypersensitivity on acute inflammatory responses to bacterial PAMPs such as LPS, we crossed floxed PTEN mice (kindly provided by Dr. Tak Mak, Canada) with Albumin cre expressing mice (kindly provided by Dr. H. Esterbauer, Vienna). We wanted to create a PI3K hyperactive strain in particular in hepatic tissue to induce insulin hypersensitivity in these mice. We could previously show that PTEN deficiency in other cell types such as macrophages led to sustained activation of the PI3K signaling pathway [15]–[17]. This phenomenon in hepatocyte PTEN deficient mice, but also in adipose tissue targeted mice, has been shown earlier and resulted in increased glucose tolerance and enhanced insulin sensitivity [18]–[20]. PTEN^fl/fl^ Alb cre positive mice (later on referred to as PTEN hepatocyte +/+ or −/−) appeared to be normal and were born with expected mendelian frequency. However as already published by others [19], [20], their livers were vastly enlarged (Fig. 1A). When we analyzed liver PTEN mRNA levels of these animals, we observed >90% deletion efficiency. At the same time cre recombinase mRNA transcription was highly upregulated in PTEN hepatocyte −/− livers (Fig. 1B).

To determine effects of LPS on PTEN expression in the liver, we treated PTEN hepatocyte +/+ and −/− littermate mice, which were less than 12 weeks of age, with *E.coli* B111:O4 LPS for 6 h. Analysis of PTEN by tissue immune blotting showed no detectable changes in liver PTEN expression upon *in vivo* LPS stimulation in wildtype mice (Fig. 1C). Again PTEN protein was greatly reduced (to<than 10%) in PTEN hepatocyte −/− animals, which was also quantified from 3 independent experiments (Fig. 1C and D).

### PTEN Hepatocyte Deficient Mice Exhibit Increased Insulin Sensitivity and Normal Metabolic Parameters

Next we evaluated the baseline metabolic parameters cholesterol, triglycerides and glucose in plasma of 8–12 week old fasted mice (16 h; o/n). We chose young mice because we know that older mice might develop hepatocarcinomas, which would certainly influence the acute inflammatory models [19]. Moreover there is agreement in the scientific community to perform the endotoxemic shock model in young animals (less than 12 weeks).

**Figure 2:**
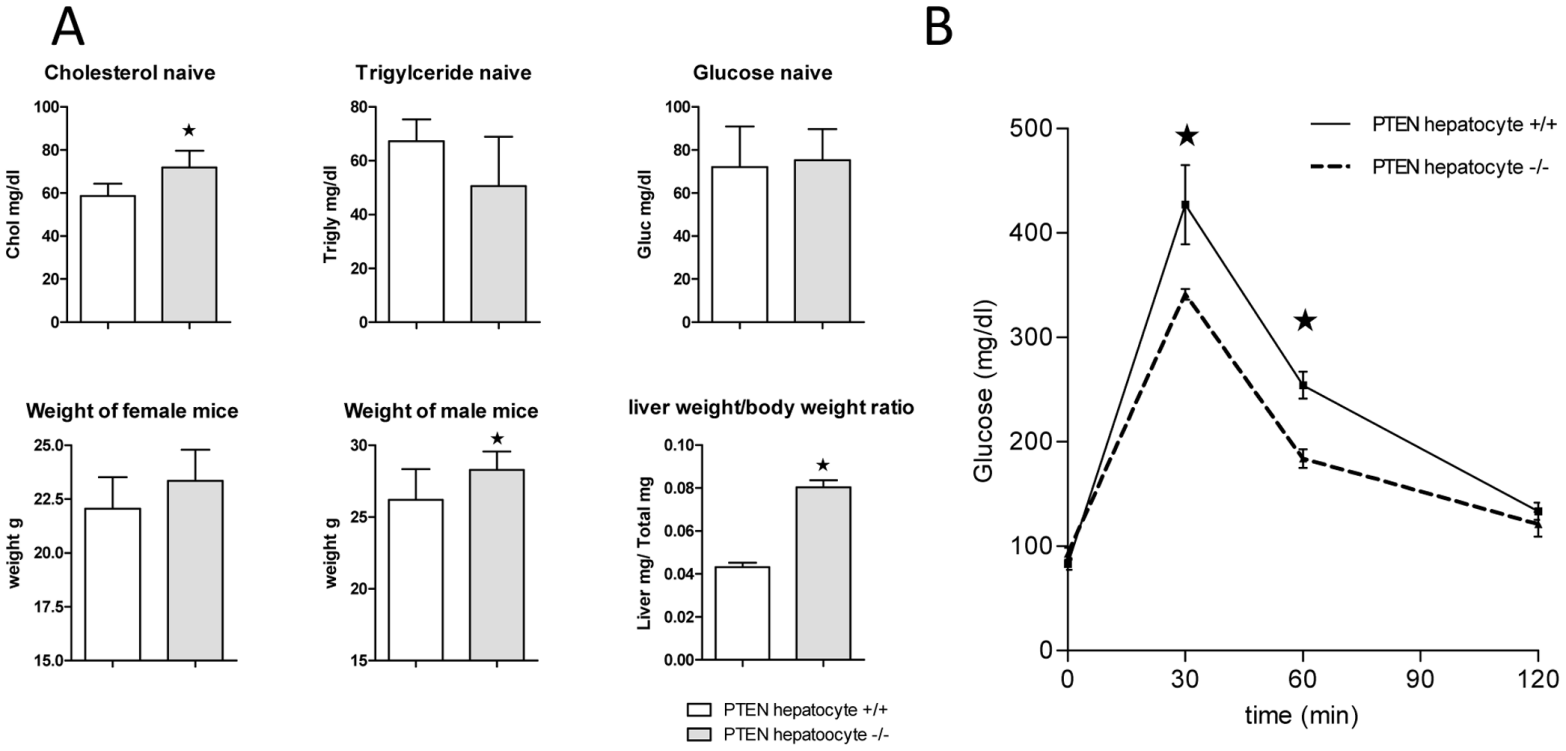
PTEN hepatocyte deficient mice exhibit increased Insulin sensitivity and normal metabolic parameters. (A) Plasma levels of metabolic parameters total cholesterol, triglycerides, and glucose were measured in 12w old naïve o/N fasted PTEN hepatocyte deficient and wildtype littermate mice (n = 5). Weight was analyzed of male and female mice (n = 10). (B) Glucose levels were measured after i.p. injection of 2 g/kg D-Glucose after the indicated time points (n = 5–7). *indicates p<0.05.

Apart from slightly but still significantly elevated cholesterol levels we did not find any overt changes in these plasma parameters, which is in line with previously published data (Fig. 2A). Although steatohepatitis was reported earlier in hepatocyte specific PTEN −/−, and increased AST and ALT levels were detected especially in older mice, we did not find alterations in these surrogate markers for liver damage (data not shown). Liver weight was dramatically increased, which is exemplified by enhanced liver/body weight ratios (Fig. 2A).

The determination insulin sensitivity in PTEN hepatocyte −/− mice was one of the most important parameters to analyze in our experimental settings. This process is a key aspect to answer the question whether sustained PI3K activity in PTEN deficient livers contributes via increased insulin sensitivity to a modulated innate immune response. Indeed we could detect a robust reduction in free plasma glucose upon intra peritoneal (i.p.) injection of 2 g/kg D-glucose into PTEN hepatocyte −/− mice and littermate controls indicating increased insulin sensitivity (Fig. 2B). These results gave us the opportunity to investigate the connection PTEN deficiency/insulin sensitivity and acute inflammatory responses.

### Insulin Sensitive, PTEN Hepatocyte Deficient Mice are Protected from Lethal Endotoxemia

To investigate the role of sustained PI3K activity and PTEN deficiency in liver tissue in acute inflammatory responses, we performed LPS induced endotoxemia in male and female mice. Usually all experiments have been performed on female mice. The only exception was the 24 h LPS survival experiment in males (Table 1).

**Table 1:** Reduced mortality in LPS challenged liver PTEN deficient male mice.

endotoxemia male, liver PTEN deficiency		
genotypes	24 h survival	
**PTEN +/+**	25,00%	3 of 12
**PTEN −/−**	61,54%	8 of 13

Liver PTEN deficient mice and littermate controls were subjected to lethal LPS challenge (15 mg/kg). Survival was monitored for 24 h.

The in vivo survival experiments were conducted with high n-numbers (male n = 25, female n = 24) as littermate controlled experiments. LPS was injected i.p. at a LD100 dose of 15 mg/kg and mice were followed closely for up to 48 h post LPS injection.

To investigate potential long term effects of PTEN hepatic gene ablation we introduced low dose endotoxemia (10 mg/kg) and observed the mice closely for up to 96 h.

**Figure 3:**
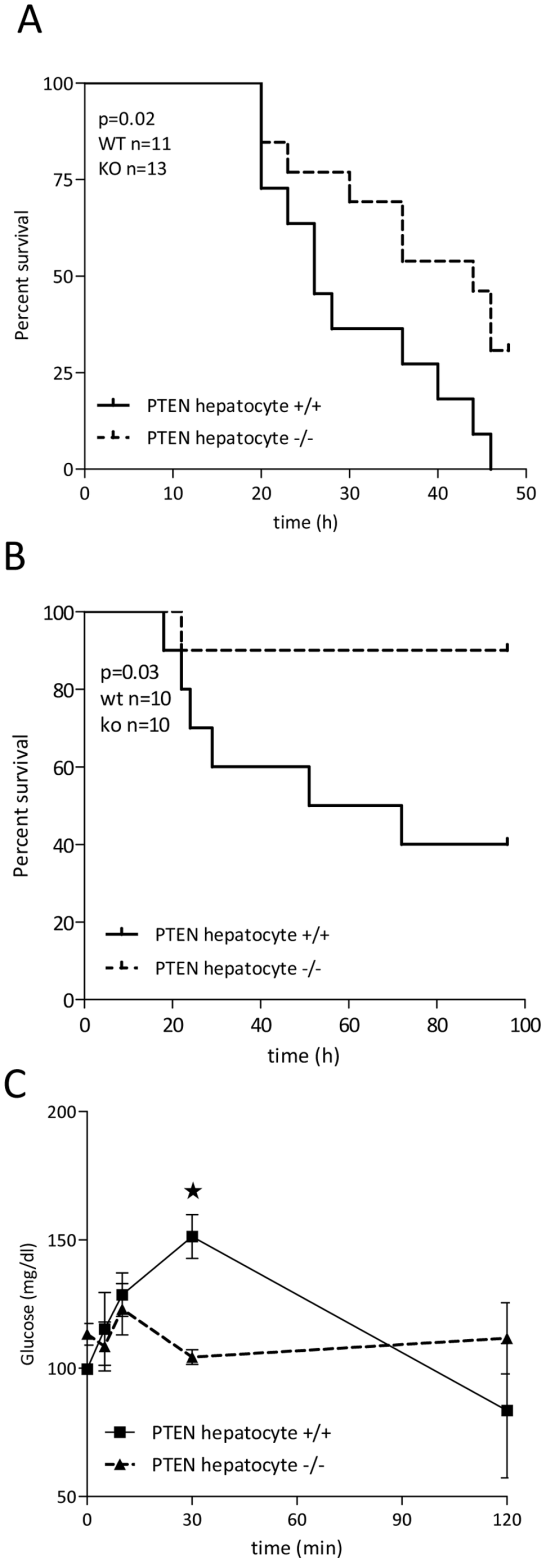
PTEN hepatocyte deficient mice are protected from lethal endotoxemia. Lethal endotoxemia (LPS: 15 mg/kg) as well as low dose endotoxemia (LPS: 10 mg/kg) was conducted on PTEN hepatocyte deficient and wildtype littermate mice (n = 10–13). (A) Kaplan-Meier/log rank survival analysis was performed of endotoxemic mice followed up for 48 h. (B) Kaplan-Meier/log rank survival analysis was performed of low dose endotoxemic mice followed up for 96 h. (C) Analysis of glucose levels of fasted endotoxemic mice was conducted at the indicated time points. *indicates p<0.05.

Interestingly, PTEN hepatocyte −/− mice showed a gender independent improved survival (Fig. 3A, B and Table 1). In the groups of female mice we could perform survival analysis depicted as Kaplan Meier curves with significant differences between the survival curves (p = 0.02; wt n = 11, ko n = 13), providing evidence for the potent beneficial properties of increased liver PI3K signaling and insulin hypersensitivity in PTEN hepatocyte deficient mice (Fig. 3A). Furthermore we could show that 24 h post LPS injection, we detected increased survival in male hepatocyte PTEN deficient mice (wt 25% vs. ko 61.54%) as well. Additionally we found significant protection in the low dose endotoxemia as well. While wt littermate mice showed 40% survival (after 96 h observation period), hepatocyte PTEN deficient mice were almost completely protected from LPS induced septic shock (Fig. 3B). This finding was confirmed, when we analyzed clinical score as well as weight loss during this 96 h observation period (Fig. S1). Hepatocyte PTEN deficient mice showed decreased signs of morbidity and weight loss was significantly diminished beyond 48 h post LPS injection.

Acute inflammatory responses in sepsis are known to cause insulin resistance [21]. Therefore we wanted to investigate the potential improvement of hyperglycemia in endotoxemic liver PTEN deficient mice. Although hyperglycemia is not a hallmark of the endotoxemic shock we could detect significant increase (up to 150 mg/dl) in plasma glucose levels 30 minutes post LPS challenge (Fig. 3C). In contrast endotoxemic PTEN hepatocyte deficient mice did not show increased glucose levels at this time point. Furthermore we did not observe a rapid decline in glucose levels, which indicate the progressing endotoxemic shock, resulting in undetectable levels 6–8 hours post LPS challenge in wildtype animals. One of the reasons for the shock like symptoms is very low plasma glucose. However the ongoing “cytokine storm” induced by bacterial LPS is actually more important for the disease progression [22].

**Figure 4:**
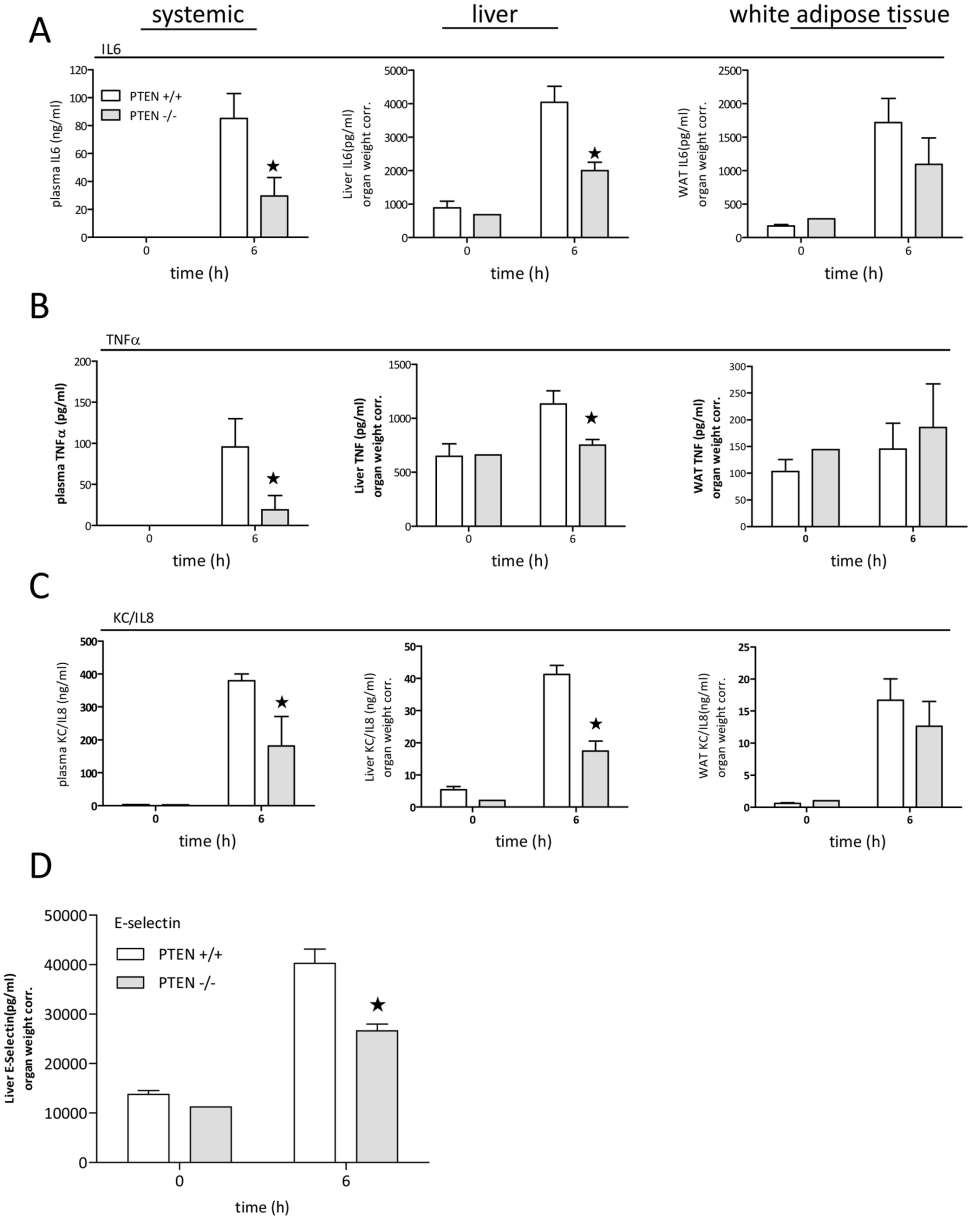
Endothelial cell activation is reduced in PTEN hepatocyte deficient mice. Lethal endotoxemia (LPS: 15 mg/kg) was conducted on PTEN hepatocyte deficient and wildtype littermate mice. Blood was drawn 6 h post LPS challenge. (A,B,C) Cytokine levels (IL6, TNFα, KC/Groα) of endotoxemic mice have been measured from plasma derived 6 h post LPS challenge by ELISA. (D) E-selectin levels were measured by ELISA. *indicates p<0.05.

Thus we analyzed the expression and the release of pro-inflammatory cyto- and chemokines 6 h post LPS challenge in a different set of experiments. We determined levels in plasma (systemic), perfused liver and perfused white adipose tissue (perigonadal fat tissue). Systemic and liver levels for IL6 (Fig. 4A), TNFα (Fig. 4B) and KC (Fig. 4C) were significantly reduced in PTEN hepatocyte −/− mice as compared to wildtype littermates. On the other hand adipose tissue specific cytokine levels were not significantly differentially regulated. Taken together, these results indicate that insulin hypersensitive PTEN hepatocyte deficient mice are protected in endotoxemia through normal glucose homeostasis and reduced inflammatory cytokine production.

To investigate vasculo-protective effects of insulin-sensitivity in murine endotoxemia, we analyzed the expression and the release/shedding of E-selectin/CD62E in challenged animals. In line with the reduced inflammatory burden we observed in endotoxemic hepatic PTEN deficient mice, we found reduced levels of E-selectin the livers of these animals as well (Fig. 4D).

### Hepatocyte Specific PTEN Deletion Protects from Peritoneal Sepsis

**Figure 5:**
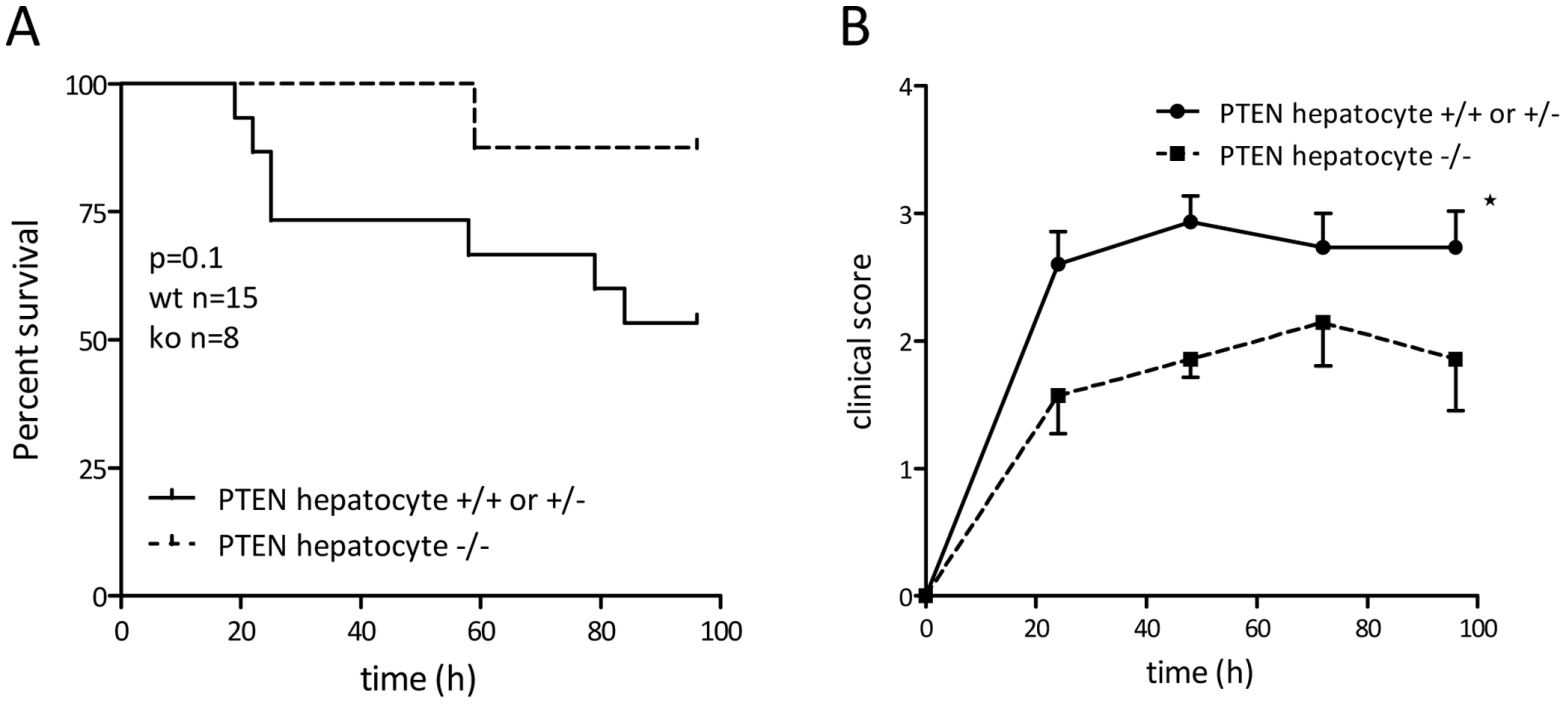
PTEN hepatocyte deficient mice show reduced mortality in CLP. Cecal ligation and puncture induced sepsis was performed with PTEN hepatocyte deficient and wildtype/heterozygous littermate mice. (A) Kaplan-Meier/log rank survival analysis was performed of CLP induced septic mice followed up for 96 h. (B) Clinical scoring was performed daily of CLP induced septic mice for 96 h.

In addition to the septic shock model using LPS *in vivo* we wanted to use a clinically relevant model of sepsis. The cecal ligation and puncture (CLP) model of peritoneal sepsis reflects several hallmarks of sepsis [23], [24]. The model we chose was a mild, CLP induced peritonitis with only one puncture (21G) of the ligated cecum. Indeed survival analysis revealed that mortality was below 50% after 96 h observation period. Interestingly hepatocyte PTEN deficient mice seemed to be protected from this polymicrobial sepsis, although it did not reach statistical significance (p = 0.1) (Fig. 5A). When we analyzed the clinical score of these septic mice we found a significant, beneficial effect in mice lacking PTEN in hepatocytes (Fig. 5B). These data indicate that even under conditions of polymicrobial infection and peritoneal as well as systemic inflammation sustained PI3K activation and insulin-hypersensitivity protect from sepsis.

### Pharmacologic Inhibition of the PI3K Signaling Pathway in Liver PTEN Deficient Mice Abrogated the Reduced Cytokine Levels

To confirm the PI3K dependent effects we observed in PTEN hepatocyte deficient mice, we conducted experiments to block the PI3K pathway in endotoxemic mice *in vivo* with the fungal metabolite wortmannin. Wortmannin (concentration of 0.06 mg/kg; i.v.) was injected into PTEN hepatocyte deficient mice and littermate controls 2 h before LPS challenge. Concerning the toxicity, we could previously show that wortmannin injection at very low doses was well tolerated and effectively reduced PI3K pathway activation in vivo [11], [12].

**Figure 6:**
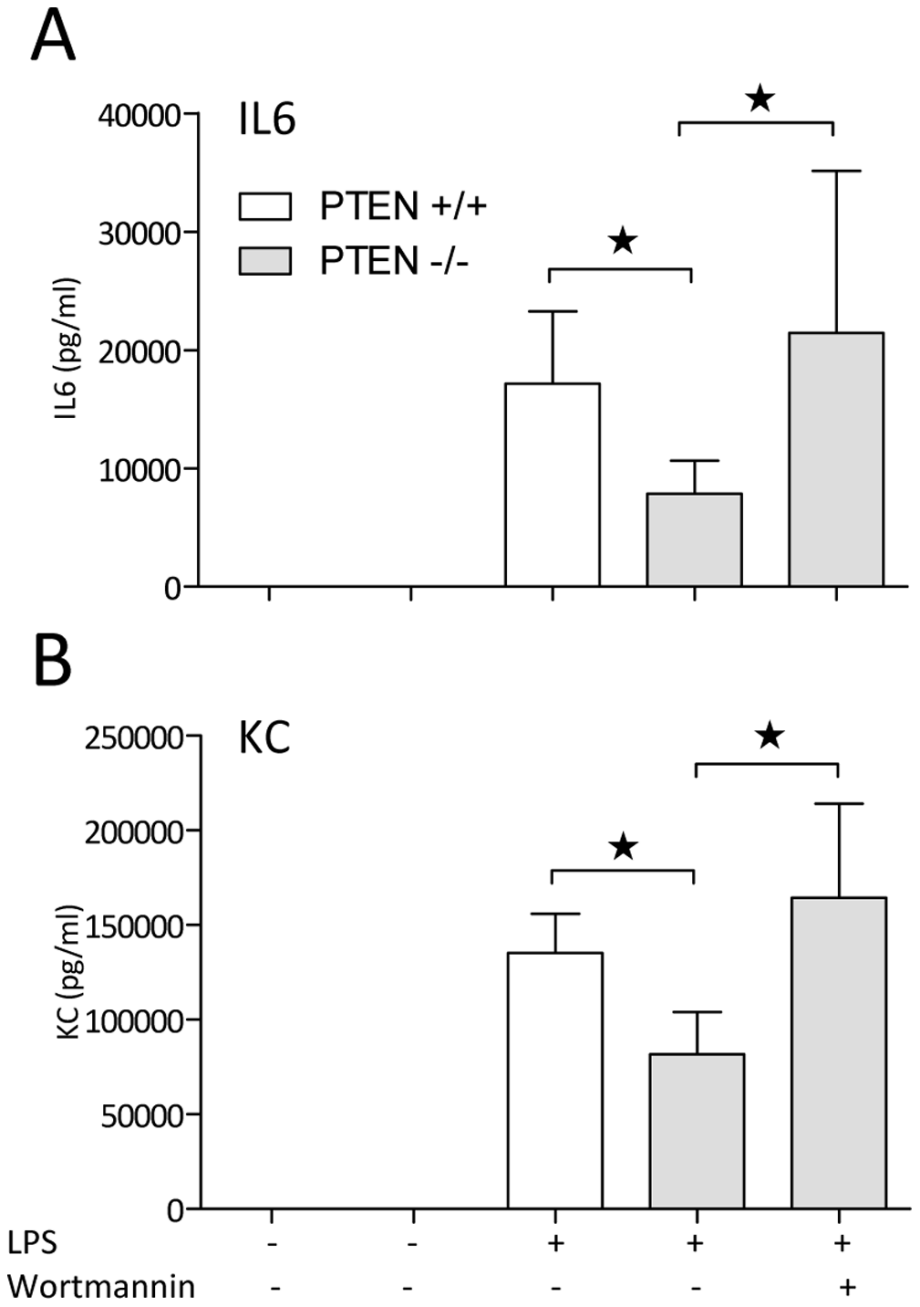
Reduced cytokine production in PTEN deficient insulin hypersensitive mice is mediated by PI3K. Lethal endotoxemia (LPS: 15 mg/kg) was conducted on PTEN hepatocyte deficient and wildtype littermate mice that were 2 h pretreated with the PI3K inhibitor wortmannin (0.06 mg/kg). Cytokine measurement by ELISA was performed on samples derived from endotoxemic mice 6 h post LPS challenge. *indicates p<0.05.

To investigate the PI3K inhibition in endotoxemic mice systemic plasma cytokine levels were measured 6 h post LPS challenge. Again we detected reduced IL6 and KC levels in the hepatocyte PTEN deficient endotoxemic animals and indeed we found that the PI3K inhibitor wortmannin could abrogate the beneficial phenotype observed in PTEN hepatocyte deficient mice (Fig. 6A and B). Moreover, we conducted endotoxemia experiments with animals lacking p85α, which is the regulatory subunit of a fully functional PI3K complex. These animals are obtained from S. Koyasu and still express the alternatively spliced isoforms p50 and p55 PI3K subunits and are therefore viable [25]. Nevertheless these mice exhibit strongly reduced PI3K activity in all organs and tissues. Therefore they are of limited use to us. However we specifically asked whether the beneficial effects we observed in liver tissue in PTEN hepatocyte deficient mice are inverted in livers of mice with the p85α −/− background. Indeed we found significantly enhanced liver IL6 levels in perfused tissue 6 h after LPS challenge (Fig. S2). Taken together these data indicate that PI3K indeed is responsible for the protective phenotype in PTEN hepatocyte deficient mice.

### Upregulation of Anti-inflammatory Genes in Livers of PTEN Hepatocyte −/− Mice

**Figure 7:**
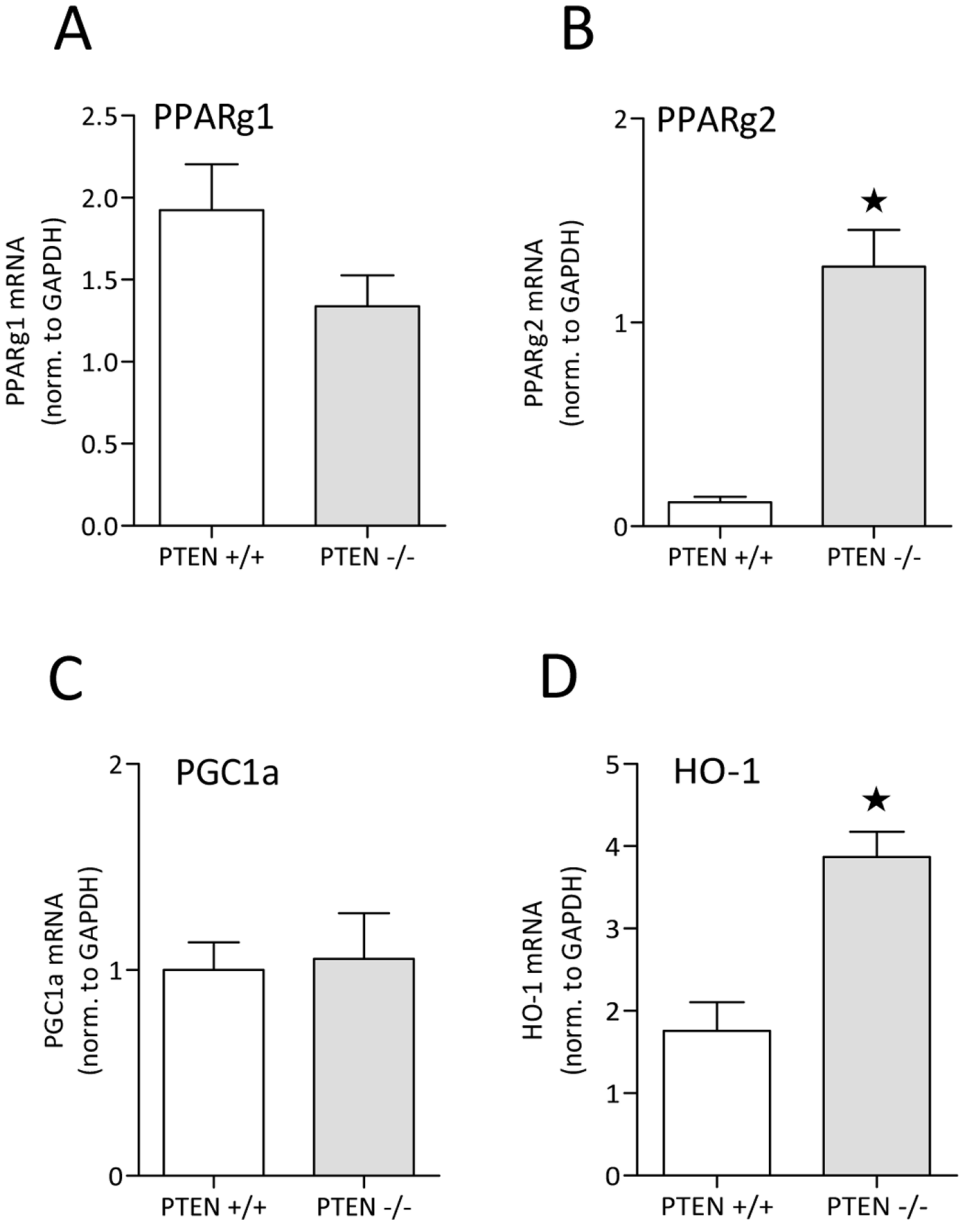
Gene expression analysis revealed upregulation of potent anti-inflammatory genes in livers of PTEN hepatocyte deficient mice. Liver mRNA was extracted from naïve mice. After reverse transcription, cDNA was analyzed by quantitative PCR for PPARγ, PGC1a and HO-1. *indicates p<0.05.

In order to discover potential molecular mechanisms how hepatic tissue and hepatic PTEN-mediated insulin hypersensitivity could contribute to the reduced inflammatory host response to LPS, we screened for the differential transcription of potential beneficial target genes in the livers of gene-targeted mice. It was already published that PPARγ was constitutively upregulated in liver-specific PTEN deficient mice [19]. This phenomenon results in enhanced adipogenesis and enlarged livers, finally resulting in severe steatosis as mentioned above. We investigated the expression of two PPARγ isoforms (Fig. 7A, B). One of which, PPARγ2, was highly upregulated on mRNA level in PTEN hepatocyte deficient livers. We found a >10-fold increase in gene targeted livers (Fig. 7B). A coactivator of this nuclear receptor, PGC1a was not affected in liver targeted mice (Fig. 7C). Interestingly we detected a more than 2-fold upregulation of the prominent macrophage marker F4/80 on mRNA level (data not shown). This might indicate that liver steatosis attracts macrophages to the organ or leads to the activation of resident cells. Although this might indicate a pro-inflammatory state of the *pten* gene targeted organ we could not detect upregulation of inflammatory in non-challenged animals. In contrast we found significant upregulation of a potent anti-inflammatory gene heme oxygenase 1 (HO-1) (Fig. 7C), which has been shown to be regulated by PPARs [26].

## Discussion

Insulin resistance and concomitant hyperglycemia are important hallmarks of sepsis. In sepsis the dysregulated host inflammatory response by the exuberant release of cytokines in particular TNFα into the circulation worsens the metabolic state of septic patients leading to metabolic alterations such as transient type II diabetes [4]. Intensive insulin treatment on the other hand has been proven to be effective in critical care patients admitted to ICUs [13]. Insulin is a strong activator of PI3K. Several publications, including our own work, point at an important role for the PI3K signaling pathway in the regulation of pro-inflammatory processes mediated by pattern recognition receptors [27]–[29].

Recently we could show that PTEN deficiency in myeloid cells reduced inflammation and protected from pneumococcal pneumonia [17]. In the current study we provide evidence for a protective effect of PI3K sustained activation/PTEN deficiency, which is not only restricted to innate immune cells but might also be relevant in liver tissue, which is thought to be an inflammation/infection sensitive organ by the induction of acute phase proteins. Here we show that early events in the endotoxemic shock model as well as peritoneal sepsis are affected by insulin sensitive hepatic tissue as well. We can show that insulin hypersensitivity in mice induced by the cell type-specific deletion of PTEN in hepatic tissue reduces the dysregulated inflammatory response upon endotoxin challenge as measured by reduced cytokine release in plasma and liver tissue. As a result the insulin hypersensitive mice appeared to be protected from LPS and polymicrobial sepsis-induced mortality.

Loss of PTEN in hepatocytes is accompanied by the loss of regulated inhibition of the PI3K signaling pathway [19]. The PI3K signaling pathway is indispensable for the transduction of insulin signals in all insulin sensitive tissues such as muscle, adipose and hepatic tissue [10]. On the other hand the concept that PI3K signaling limits innate immune signaling [27], supports the idea that hepatic PI3K activity might contribute to protective features of insulin observed in critically ill patients. Indeed we could show that PTEN deficiency in liver tissue reduced the inflammatory burden during endotoxemia in very early events within 6 h post LPS challenge (Fig. 4). Furthermore we could provide evidence that even under infectious conditions, using the CLP sepsis model, hepatic PTEN deficiency showed a trend for protection (Fig. 5). This protective effect goes in parallel with increased insulin hypersensitivity in naïve mice (Fig. 2B). Hyperglycemia is known to contribute to inflammation, vascular activation/dysfunction and metabolic alterations in septic conditions [4]. Our data support the notion that in the model of insulin hypersensitivity by hepatic PTEN ablation, glucose metabolism in lethal endotoxic shock is normalized (Fig. 3C). We did not observe this slight but significant increase in glucose levels up to 150 mg/dl (in sepsis glucose levels may rise 2–3-fold higher) in fasted PTEN −/− animals before the animals go into metabolic shock and glucose levels drop below detectable levels (6–8 h post LPS challenge <20 mg/dl; for comparison in non-fasted animals see [12]). However it is still unclear whether the protective effect is caused by the reduced glucose levels. Glucose does not rise to levels that would be considered harmful for the animals (during GTT glucose levels rise three times higher as compared during endotoxemia, albeit without the inflammatory burden). Furthermore in septic patients as well as in animal models of sepsis glucose level reach much higher plateau levels as compared to murine endotoxemia. Another likely possibility is that insulin sensitivity indirectly affects the acute inflammatory host response. Probably the insulin sensitive liver in PTEN deficient animals generates second messenger molecules that render the host unsusceptible to LPS induced shock. One of those targets is supposed to be carbon monoxide (CO). We found that PPARγ is highly upregulated in livers of PTEN deficient mice. PPARγ itself is an anti-inflammatory transcription factor known to interfere with a variety of pro-inflammatory signaling cascades. There is wealth of published data proving the effective competition of nuclear receptors such as PPARs for transcriptional co-activators and pro-inflammatory TFs such as NFκB, AP-1 or EGR-1 [30]–[33]. Also other PPARs have been shown to be protective in acute inflammatory diseases [34]. In addition to the direct anti-inflammatory properties of PPARs, the observation was made that PPARs efficiently regulate the transcriptional activation of the *heme oxygenase 1* gene (HO-1) [26]. HO-1 has been shown to be upregulated in livers of rats infused with LPS (3 mg/kg) [35]. HO-1 is part of the hemoglobin degradation system resulting in the generation of anti-inflammatory carbon monoxide and bilirubin. Bilirubin proved to be protective in murine endotoxemia [36]. CO on the other hand inhibited LPS-mediated inflammatory responses of macrophages [33]. Interestingly there is a connection between PPARγ and carbon monoxide as well. It has been shown that CO leads to modification of PPARγ with increased activity supporting the anti-inflammatory properties of CO [37]. We speculate that in a cyclic process PPARγoverexpressed in insulin-sensitive PTEN deficient livers upregulates HO-1. Thereby HO-1 produces excess amounts of bilirubin and CO by the conversion of hemoglobin. CO in turn activates and stimulates PPARγ, reducing effectively the harmful inflammatory response.

Taken together our data indicate that insulin hypersensitivity by reduced expression of PTEN in livers of gene targeted mice resulted in reduced inflammatory burden for the host and protection form endotoxic shock and polymicrobial sepsis through upregulation of the anti-inflammatory molecules PPARγ and HO-1.

## Supporting Information

Figure S1 **PTEN hepatocyte deficient mice show improved clinical score and reduced weight loss in low dose endotoxemia.** Low dose endotoxemia (LPS: 10 mg/kg) was conducted on PTEN hepatocyte deficient and wildtype littermate mice (n = 10). (A) The clinical score of endotoxemic mice was assessed and (B) weight reduction was determined up to 96 h post LPS challenge.
(TIF)

Figure S2 **Upregulation of hepatic IL6 in p85α gene deficient endotoxemic mice.** Endotoxemia (LPS: 15 mg/kg) in p85α/PI3K deficient mice was induced and livers were isolated 6 h post LPS challenge. IL6 levels in liver homogenates was determined by ELISA.
(TIF)
