# Supplementary figures and images for: Insulin Hypersensitivity Induced by Hepatic PTEN Gene Ablation Protects from Murine Endotoxemia
Source: PLoS One. 2013 Jun 25;8(6):e67013. doi: 10.1371/journal.pone.0067013 (PMC3692528; doi:10.1371/journal.pone.0067013)

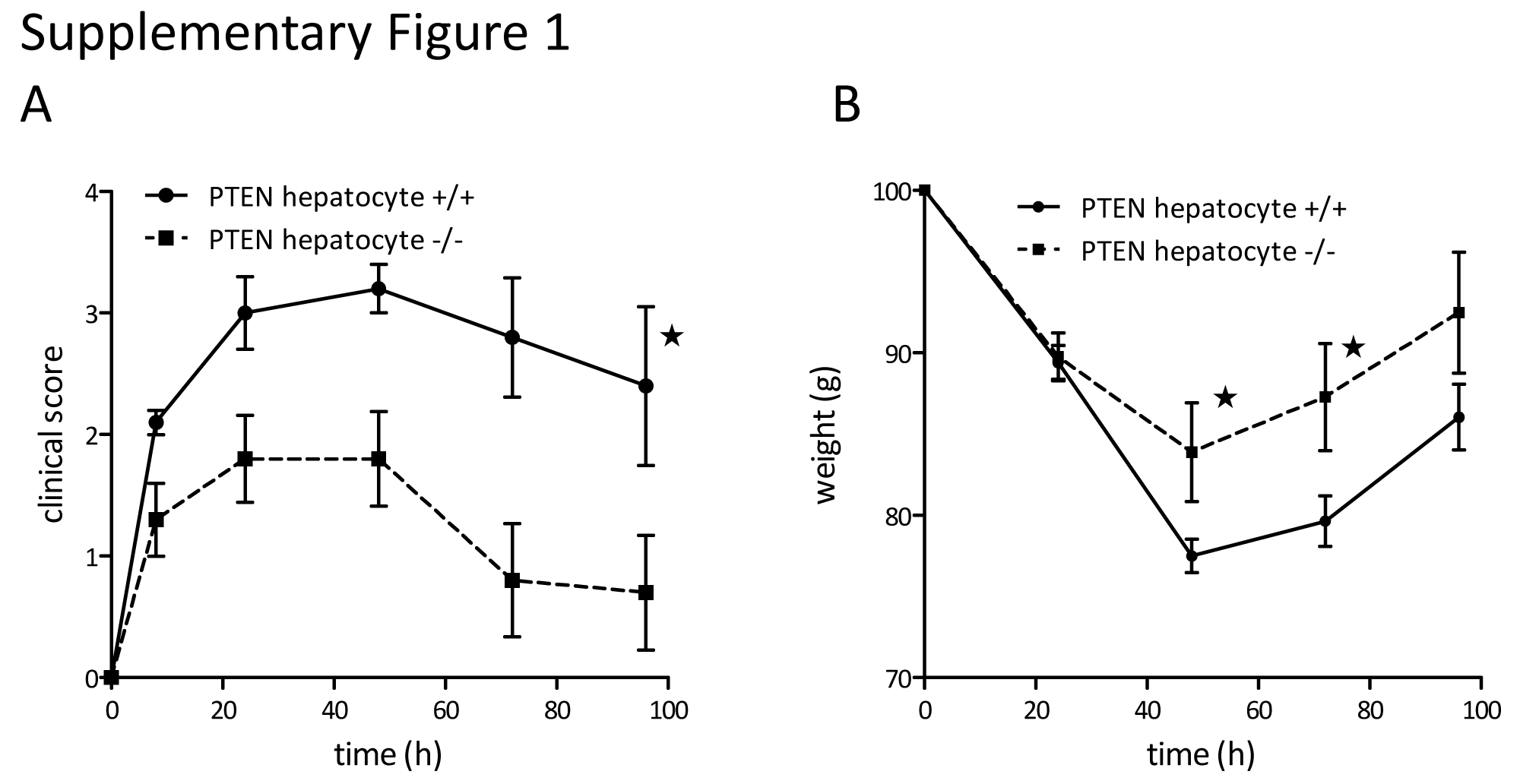

Supplement: Figure S1 — PTEN hepatocyte deficient mice show improved clinical score and reduced weight loss in low dose endotoxemia. Low dose endotoxemia (LPS: 10 mg/kg) was conducted on PTEN hepatocyte deficient and wildtype littermate mice (n = 10). (A) The clinical score of endotoxemic mice was assessed and (B) weight reduction was determined up to 96 h post LPS challenge. (TIF) [file pone.0067013.s001.tif]

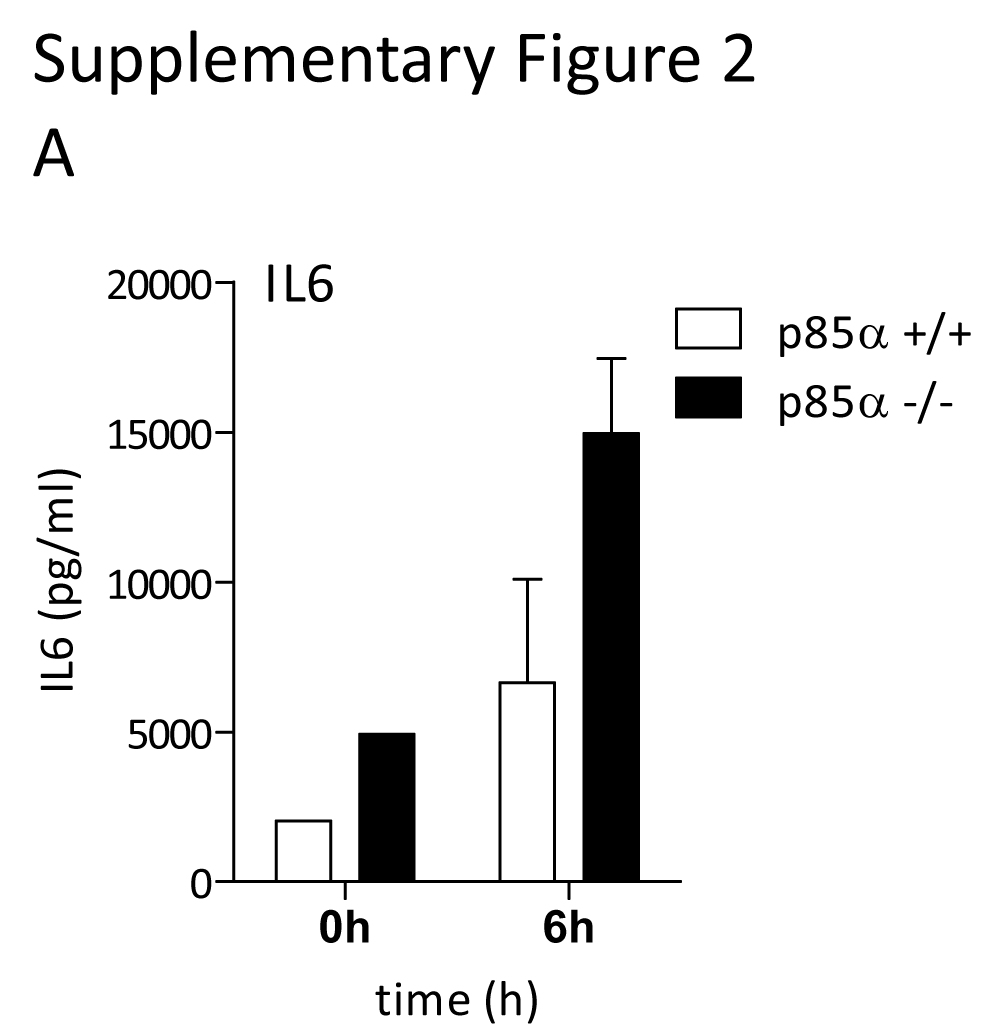

Supplement: Figure S2 — Upregulation of hepatic IL6 in p85α gene deficient endotoxemic mice. Endotoxemia (LPS: 15 mg/kg) in p85α/PI3K deficient mice was induced and livers were isolated 6 h post LPS challenge. IL6 levels in liver homogenates was determined by ELISA. (TIF) [file pone.0067013.s002.tif]
